# Manipulator Control System Based on Flexible Sensor Technology

**DOI:** 10.3390/mi14091697

**Published:** 2023-08-30

**Authors:** Jian Chen, Chunfang Wang, Jingxin Chen, Binfeng Yin

**Affiliations:** School of Mechanical Engineering, Yangzhou University, Huayangxi Road No. 196, Yangzhou 225127, China; jian.chen@yzu.edu.cn (J.C.); mz120220893@stu.yzu.edu.cn (C.W.); mathematical202207@163.com (J.C.)

**Keywords:** manipulator control, flexible sensor, manufacturing process, gesture recognition, EMG control, data gloves

## Abstract

The research on the remote control of manipulators based on flexible sensor technology is gradually extensive. In order to achieve stable, accurate, and efficient control of the manipulator, it is necessary to reasonably design the structure of the sensor with excellent tensile strength and flexibility. The acquisition of manual information by high-performance sensors is the basis of manipulator control. This paper starts with the manufacturing of materials of the flexible sensor for the manipulator, introduces the substrate, sensor, and flexible electrode materials, respectively, and summarizes the performance of different flexible sensors. From the perspective of manufacturing, it introduces their basic principles and compares their advantages and disadvantages. Then, according to the different ways of wearing, the two control methods of data glove control and surface EMG control are respectively introduced, the principle, control process, and detection accuracy are summarized, and the problems of material microstructure, reducing the cost, optimizing the circuit design and so on are emphasized in this field. Finally, the commercial application in this field is explained and the future research direction is proposed from two aspects: how to ensure real-time control and better receive the feedback signal from the manipulator.

## 1. Introduction

Robots have gradually played an important role in machine manufacturing, electric power, the nuclear industry, metallurgy, light industry, and other fields. As a kind of mobile robot, the remote control of manipulators has been widely studied by scientists; a manipulator is a device that can imitate the actions of human hands to grasp, carry objects, or operate tools [1]. In order to achieve these functions, a system is usually used to control the operation of the manipulator, which is called the manipulator control system, and generally includes flexible electronics (for data acquisition), computer processing modules (for data processing), and transmission modules (for signal transmission). Through this control system, to complete the remote operation of the manipulator, the use efficiency of the manipulator can be improved. Traditional remote control can be based on machine-vision technology, which captures people’s hand movements through the camera, uploads the images or videos to the computer for analysis and processing, obtains the hand movement information, and sends it to the manipulator to perform corresponding actions, and completes gesture recognition and tracking [2,3,4]. However, the problem with this method is that it is greatly affected by environmental factors such as light. Compared with the control method based on vision technology, the control method based on flexible sensor technology through a data glove [5] or through electromyography (EMG) [6] is more widely used. This method has higher detection accuracy and a stable information collection process.

Of course, this is related to the development of flexible sensors. A flexible sensor is one of the basic components of wearable devices. It is made of various flexible materials, with skin friendly, flexible, and comfortable characteristics, and it is a sensing device that can collect the measured information and convert the measured information, such as the large amount of elastic strain [7], etc., into electrical signals or other signals. Due to the continuous development of manufacturing materials, the continuous progress of manufacturing processes, and the increasing maturity of flexible sensor technology, the flexible sensor designed for manipulator control has higher sensitivity and stronger stability. In view of this, the use of wearable flexible sensors to collect information from the human hand and control the movement of the manipulator has become a hot research topic. It is widely used in fruit and vegetable picking, medical rehabilitation, assistance for the elderly and disabled, and hazardous electrical work.

**Figure 1:**
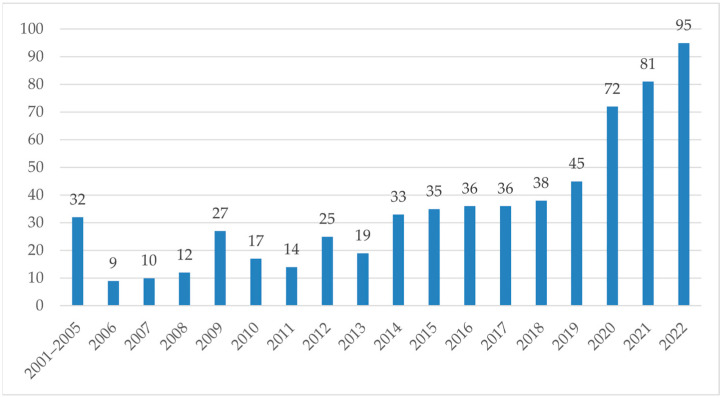
Literature growth trend of manipulator-control research using flexible sensors over the years.

Using the Web of Science (WOS) database to search the literature using the keywords “flexible sensing technology” and “gesture recognition”, we find that there are related papers published every year and the number of papers is increasing. The literature growth trend of manipulator-control research based on flexible sensor technology over the years is shown in Figure 1. In the early stage, due to the immaturity of flexible sensing technology, the previous research mainly focused on machine-vision technology or the combination of flexible sensors and machine vision for gesture recognition, resulting in a small number of related research papers. In later stages, research on flexible sensing technology made some progress due to the continuous development of multidisciplinary fields, such as digital communication and artificial intelligence. Relevant interdisciplinary studies continue to emerge, eventually leading to a sharp increase in the amount of literature in this field.

**Figure 2:**
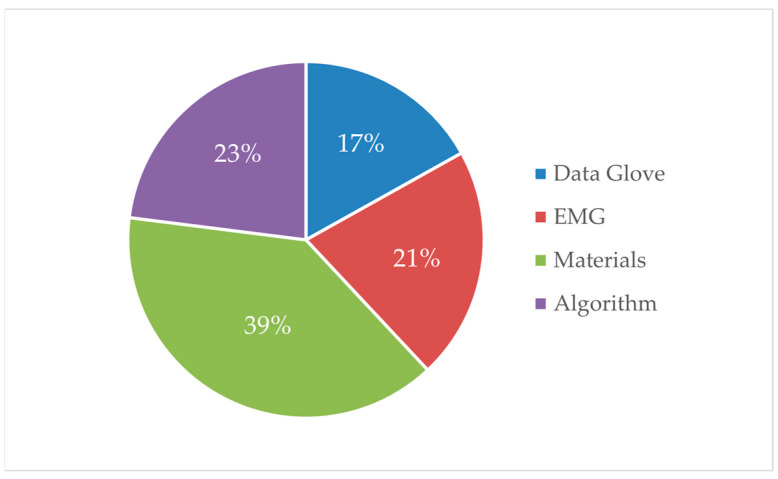
Proportion of the amount of the literature searched by different keywords.

By searching the literature using the keywords “flexible sensing technology” and “gesture recognition” and adding the keywords “material”, “data glove”, “myoelectric”, and “algorithm”, a literature proportion distribution diagram can be obtained as shown in Figure 2. It can be seen from Figure 2 that 39% of the literature focuses on materials for manufacturing flexible sensors. This indicates a significant interest in how materials affect sensor performance. In addition, 23% of the literature is dedicated to algorithmic research by optimizing existing algorithms or creating new ones to address issues such as control and detection accuracy. However, there is no significant difference in the number of studies on the two different control methods, indicating that the search for optimal control methods is ongoing. However, recognition difficulty, sensitivity, recognition accuracy, and stability determine the quality of the control method. In order to solve these problems, relevant research is mainly carried out by improving the design scheme, upgrading the preparation materials, and optimizing the process flow. Through the design of the microchannel and microstructure, a flexible sensor with higher sensitivity, faster response, and more stable operation will be produced. It will also strengthen the research of some preparation materials and improve the control sensitivity and stability of flexible sensors by improving the properties of materials. The process directly determines the quality of design products and the exploration, improvement, and promotion of the process level is also an important issue that needs continuous research.

According to the results of the literature search, we found that, although many scholars have done a lot of research in this area in recent years, there are few literature reviews on robot control systems based on flexible sensor technology and they are concentrated in the last five years. Xu et al. [8] summarized the progress of flexible sensors that can be used for gesture recognition interactions, focusing on the performance of flexible devices constructed using functional nanomaterials. Similarly, the review [9] specifically summarized the performance, fabrication, and application of flexible sensors made of carbon nanotubes (CNTs). Yin et al. [10] detailed the mechanism of designing various flexible sensors based on different mechanisms and material-selection strategies but lacked a specific description of the gesture–recognition interaction process. Pan et al. [11] summarized the number and changing trend of literature on data-glove technology but did not include the content of recognition control using an EMG signal. Si et al. [12] believed that accurate gesture recognition can be achieved by placing sensors on fingers or integrating them into data gloves, but did not summarize the recognition process.

Therefore, according to the different research focuses emphasized by different review papers, this paper decides to focus on the relevant contents of materials, processes, and control methods, and summarize the sensor integration and accuracy of different methods. The main contributions mainly include the following aspects:

(1) This paper provides a comprehensive summary of research on the flexible sensor technology of a manipulator control system, which includes manufacturing materials, manufacturing processes, and control methods;
(2) This paper provides an overview of two related sensor information acquisition technologies: direct acquisition of EMG signals from skin surfaces and integration of gesture change information into data gloves;
(3) To summarize the existing problems from the three aspects of material, process, and control, respectively, and to provide a reference for future research in this area from a commercialization perspective.

The remaining contents of this paper are as follows: the second part mainly introduces the components of the flexible sensor, the types of manufacturing materials of each module, and the manufacturing process of the flexible sensor; the third part introduces two different types of manipulator control based on flexible sensor technology; and the fourth part summarizes the problems from different aspects. Finally, the paper summarizes the whole text and looks forward to the future research on manipulator control.

## 2. Materials and Processes for Fabricating Flexible Sensors

Comfort, stretchability, skin affinity, and micro are the important characteristics of wearable flexible sensors. The selection of flexible materials with high stretchability and good electrical conductivity is crucial for flexible sensors. Different flexible sensors use different materials but the whole consists of three components: substrate material, sensing material, and flexible electrode. There are many methods for manufacturing flexible sensors, such as dipping and coating, lithography, inkjet printing, screen printing, etc. The following is a brief introduction to the two aspects of materials and processes.

### 2.1. Materials

**Figure 3:**
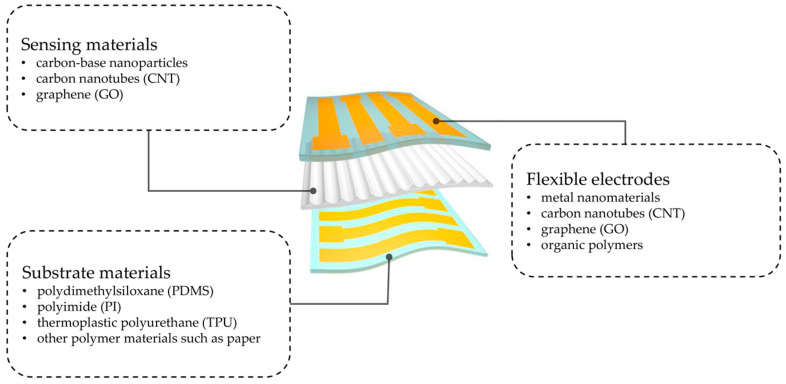
Component modules of the flexible sensor and the materials commonly used in each module. Part of the figure is reprinted from [13], copyright (2019) with permission from ACS Publications.

As shown in Figure 3, commonly used substrate materials are mainly polydimethylsiloxane (PDMS), polyimide (PI), thermoplastic polyurethane (TPU), and other polymer materials such as paper; sensing materials are carbon-based nanomaterials (CBN), such as carbon-based nanoparticles, carbon nanotubes (CNT), and graphene (GO); and conductive materials are generally carbon nanomaterials, metal nanomaterials, organic polymers, and so on.

#### 2.1.1. Substrate Material

The flexible substrate is an indispensable part of the flexible pressure sensor. In order to meet the requirements of the flexible sensor, the properties of flexibility, corrosion resistance, stability, thinness, and light have become the key indicators for the selection of the flexible substrate. The main parameters that determine these indices include the thermal expansion coefficient (TE), moisture barrier property (MBP), radius of curvature (ROC), degradability (DA), and Young’s modulus (YM). Commonly used substrate materials are mainly polydimethylsiloxane (PDMS) [14,15,16,17], polyimide (PI) [18,19], thermoplastic polyurethane (TPU) [20,21,22,23,24,25], and other polymer materials such as paper, each of which has great application potential in the fabrication of flexible sensors with different functions. The physical properties and advantages/disadvantages of common substrate materials are compared in Table 1.

**Figure 4:**
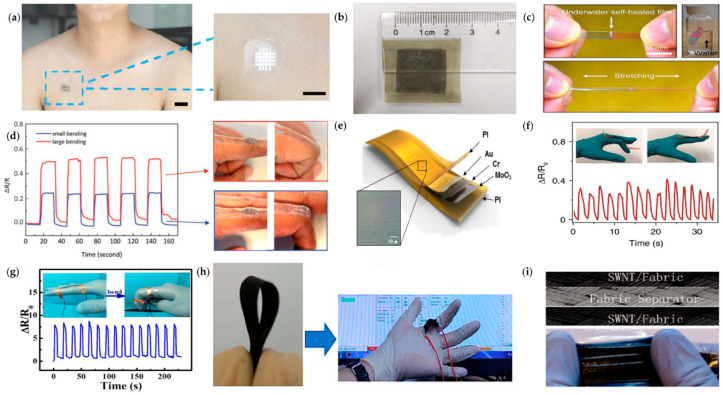
Research examples of sensors based on PDMS substrates, in which (**a**) is the photograph of a flexible sensor attached to a human chest to measure respiratory rhythms, reprinted from [13], copyright (2019), with permission from ACS Publications. (**b**) shows the triboelectric tactile sensor based on wrinkled PDMS/MXene composite films, reprinted from [30], copyright (2020), with permission from Elsevier. (**c**) shows the self-healing of the PDMS-MPU0.4-IU0.6 film, which can take place even underwater, reprinted from [31], copyright (2018), with permission from Wiley. (**d**) shows the performance of the self-adhesive strain sensor on a finger, reprinted from [16], copyright (2016), with permission from Wiley. (**e**) is the schematic illustration of the polyimide (PI) encapsulated crack sensor, reprinted from [31], copyright (2018), with permission from Applied Sciences. (**f**) shows the sensing application of the TPU/CB strain sensor in human motion, reprinted from [33], copyright (2021), with permission from PubMed Central. (**g**) is the photograph of the CNT/TPU strain sensor attached to the index finger, reprinted from [34], copyright (2019), with permission from ACS Publications. (**h**) is the photograph of human-motion monitoring with the strain sensor, reprinted from [35], copyright (2017), with permission from ACS Publications. (**i**) are the optical images of the cured film before and after stretching, reprinted from [36], copyright (2010), with permission from ACS Publications.

PDMS, as the most commonly used flexible substrate material, has been widely used by researchers in electronic skin, flexible circuits, superhydrophobic surfaces, microfluidic chips [26,27,28], and other fields due to its easy availability, low modulus, strong mechanical compliance, nontoxicity, extremely high tensile property, good biocompatibility, and, most importantly, customizable surface chemistry [29]. Ordinary PDMS elastomers have limited stretchability, adhesion, and flexibility, and can be improved to have better properties by some physical or chemical treatment of the material. However, the main disadvantage is that it is not degradable after disposal, which causes environmental pollution. By modifying the morphology of the PDMS microstructure, Zeng et al. [13] fabricated a highly sensitive flexible pressure sensor with a sensitivity of 14.26 kPa^−1^ and a response time of less than 50 ms, as shown in Figure 4a. As shown in Figure 4b, Cai et al. [30] proposed a self-powered tactile sensor based on PDMS/MXene. The best sensitivity reaches 0.18 V/Pa at 10–80 Pa and 0.06 V/Pa at 80–800 Pa, respectively, higher than most other self-powered tactile sensors, the different sensitivity under low and high pressure is related to wrinkles. It can be used to monitor complex human physiological signals, showing great potential for disease detection and health assessment. By using liquid metal eutectic gallium indium (EGaIn) as the conductive layer and PDMS-MPU0.4-IU0.6 as the encapsulation and support layer, Kang et al. [31] fabricated a new class of stretchable and autonomous self-healing electrodes, which show great improvement in toughness, stretchability, and stability, as shown in Figure 4c. Similarly, Li et al. [32] designed a new polymer chain based on PDMS by taking advantage of the fact that the iron bond is easily broken and reformed. This Fe-Hpdca-PDMS polymer shows good mechanical strength and a very high tensile property, which gives the elastomer excellent ductility and self-healing properties and can be used as a support material for self-healing artificial muscle actuators. Jeong et al. [16] demonstrated a simple method to adjust the mechanical compliance, elongation at break, and adhesion to human skin of PDMS by adding different amounts of ethoxylated polyethyleneimine (PEIE) additives to the mixture of silicone base and crosslinker of the PDMS-based elastomer and, then, fabricated a flexible electronic device for detecting finger movements, as shown in Figure 4d.

Polyimide (PI) has excellent thermal stability. Its chemical properties can remain stable in the range of −240 °C to 260 °C; although PI material is not malleable, it can be bent, has excellent mechanical properties and corrosion resistance, and is an ideal material for flexible sensor substrate. The problem with PI is that its color is yellow. This makes it highly transparent and also reduces its adsorption capacity on flexible materials. In 2018, Kim et al. [37] fabricated a nanocrack sensor on the PI substrate by a stretching method, which has a high sensitivity to strain and low response (the strain coefficient exceeds 10,000 and the response time is only 5 ms at 2% strain), and has good application potential in motion monitoring, as shown in Figure 4e.

Polyurethane (PU) can be used in the manufacture of flexible sensors, which have the properties of temperature resistance, wear resistance, high strength and elasticity, and can provide good ductility for flexible sensors. As shown in Figure 4f, Wang et al. [33] fabricated a highly sensitive strain sensor by embedding TPU fiber film in carbon black (CB) particles with the adjustable support network, which has broad application prospects in intelligent terminal, electronic skin, voice measurement, human motion monitoring, etc. Zhou et al. [34] designed a stretchable strain sensor with a cracked structure by spraying CNT ink coating on a TPU fiber mat. The sensor can detect a subtle and wide range of human motion with its excellent sensitivity and good stability, as shown in Figure 4g.

In addition, polyethylene terephthalate (PET) is also one of the commonly used polymers for manufacturing flexible sensors, it has good flexibility, excellent electrical insulation, and can maintain stable physical properties over a wide temperature range but its heat resistance is inferior to PI. Most of the substrate materials mentioned above are thermosetting resins and the flexible sensor parts made of them are difficult to recycle or degrade. With the widespread use of flexible sensors, a large amount of electronic waste will be generated. Therefore, in order to reduce the environmental impact, there is an urgent need for some renewable or recyclable materials, such as paper, as substrate. In addition, in some special applications of flexible sensors, paper, and some synthetic textiles are also a suitable choice as the base material of flexible sensors because this is likely to minimize the design complexity of the system. Liu et al. [35] fabricated a flexible and degradable paper-based strain sensor by dip-coating the paper substrate in an aqueous suspension of carbon black (CB) and carboxymethyl cellulose (CMC), as shown in Figure 4h. It has high stability and can be used to monitor various human movements. There are five major limitations of the current paper-based microfluidic system: (1) the conductors cannot be interconnected: holes must be drilled between the different layers of paper and the patterns on the different layers must be aligned so that the printed conductors can be connected between the different layers of paper; (2) the flow of water-based liquids is slowed down: conventionally printed carbon and silver electrodes are hydrophobic, which can cause their flow to slow down or stop; (3) paper surface area is limited: the viscous ink does not penetrate into the paper substrate, so the inner surface of the paper is not used; (4) poor contact adhesion and stability: The ink does not adhere well to the bare paper, and it is easily broken or torn; and (5) the blockage of the hole in the paper: after the electrode is printed on the paper, the aqueous solution cannot pass through the printed electrode. Hamedi et al. [38] demonstrated a technology fabric that overcomes these five limitations by fabricating circuits and microfluidic structures on paper. Hu et al. [36] used single-wall carbon nanotube (SWNT) inks to fabricate highly conductive textiles by an extremely simple “dip and dry” process, which exhibited excellent flexibility and stretchability, as shown in Figure 4i. Based on these flexible substrate materials, the flexible sensor has the characteristics of softness, stretchability, bendability, wearability, etc., and is widely used in smart textiles to stabilize and integrate multifunctionality beyond clothing [39].

#### 2.1.2. Sensor Material and Flexible Electrode

The materials used for the sensor and the flexible electrode are very similar. The sensing material is one of the key materials of the flexible strain sensor, which can convert the external pressure/strain signal of the sensor into an electrical signal. In recent years, researchers have generally fabricated flexible strain sensors with good mechanical properties based on CBN. CBNs include carbon-based nanoparticles, CNT, GO, etc. Due to their unique electrical properties and good piezoresistive sensitivity, CBNs are the main materials for fabricating flexible electrodes. Flexible electrodes are an important part of flexible sensors and are developed from various conductive materials. Currently, the main materials are not only CBN but also metal nanoparticles. Carbon materials have special electrical conductivity and structural diversity, overcoming the inherent performance limitations of traditional transparent electrode materials. Among metal nanoparticles, some noble metal nanoparticles or nanowires, such as gold, silver, and nickel, and nanoparticle/nanowire composites, are widely used as flexible electrode materials for wearable sensors [40,41,42,43]. In addition, there are some emerging materials, such as organic polymers, liquid metals, ionic hydrogels, etc.

**Figure 5:**
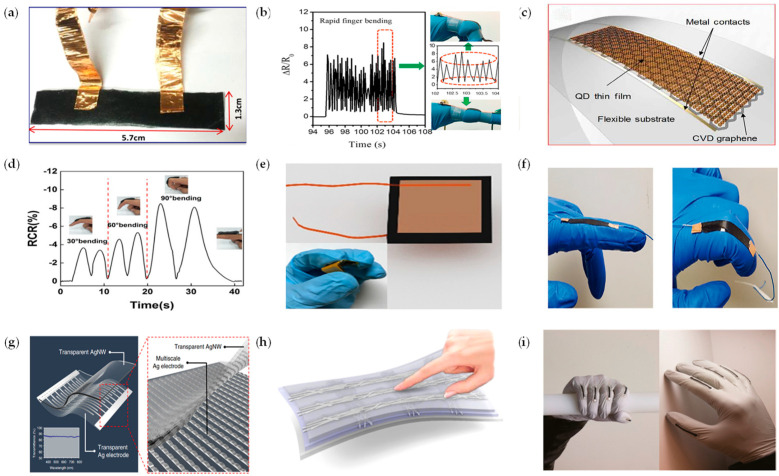
Example of a carbon nanotube-based sensor application. (**a**) is the optical photograph of the as-synthesized VACNT-PDMS thin-film sensor, reprinted from [47], copyright (2021), with permission from ACS Publications. (**b**) is the photograph of the 3D graphene films used for rapid finger bending detection, reprinted from [49], copyright (2018), with permission from Wiley. (**c**) Schematic illustration of the assembly of graphene and QDs on a flexible substrate, reprinted from [50], copyright (2019), with permission from Science Advances. (**d**) shows the relative change in sensor resistance with finger bending, reprinted from [51], copyright (2018), with permission from Elsevier. (**e**) shows the graphene paper pressure sensor with excellent sensitivity, reprinted from [52], copyright (2017), with permission from ACS Publications. (**f**) shows the photos of the strain sensor on the index finger, reprinted from [53], copyright (2020), with permission from Wiley. (**g**) shows the photograph of the schematic of the whole sensor system with an illustration of the magnified image, reprinted from [54], copyright (2019), with permission from PubMed Central. (**h**) is the photograph of the transparent and stretchable capacitive touch sensor, reprinted from [55], copyright (2017), with permission from American Chemical Society. (**i**) is the photograph of five stretchable strain sensors attached to the finger joints of the glove, reprinted from [56], copyright (2015), with permission from American Chemical Society.

Carbon nanotubes (CNT), as one of the new materials with great potential, have excellent properties, such as light weight, good conductivity, and high surface utilization due to their perfect hexagonal interconnection structure. The tube diameter of CNT is controllable, and it can be applied to the production of large-area, low-cost flexible sensors through simple processing and synthesis. The mass-produced carbon nanotubes are compatible with large-area solution processing technology; carbon nanotubes can be directly deposited on flexible materials or stretchable substrates, and are widely used as electrode materials for flexible strain and pressure sensors [44]. Lipomi et al. [45] reported a type of skin-like pressure and strain sensors based on transparent elastic films of CNTs, which can be made stretchable by applying strain along each axis and then releasing the strain. Jung et al. [46] fabricated a dry electrocardiogram (ECG) electrode based on CNT–PDMS composites, which can be easily connected to conventional ECG devices, and demonstrated its long-term wearable monitoring capabilities as well as its robustness to motion and sweat. In general, significant structural deformation of the material can achieve significant changes in the electrical signal to meet the requirements of flexible sensors for high sensitivity. On this basis, it is also necessary to maintain the integrity of the elastomer structure during large deformation to meet the tensile requirements; therefore, a possible scheme to improve the mechanical and electrical properties of CNT is to apply vertically aligned carbon nanotubes. Paul et al. [47] designed a vertically aligned carbon nanotube (VACNT)–PDMS composite structure as the sensing material of the stretchable sensor, as shown in Figure 5a. The sensor has excellent strain deformation ranging from 0.004% to 30%. Boutry et al. [48] fabricated a bionic electronic skin formed by CNT top and bottom electrodes embedded in a PU elastic substrate, which has excellent electrical stability when mechanical deformation is applied, can control the robotic arm to perform various tasks, and can measure and discriminate forward and tangential pressures in real time.

Graphene (GO) has the characteristics of being thin and transparent, having good electrical and thermal conductivity, and being a very promising flexible strain sensor material. Graphene has two preparation methods: the chemical vapor deposition method and the natural graphite stripping method. The graphene prepared by the chemical vapor deposition method [57] may have insulating impurities during the preparation process, resulting in poor electrical conductivity. Tolerable strain is usually less than 1%. The graphene prepared by the natural graphite stripping method has the advantages of large-scale production and low cost, which is more conducive to practical application [58,59,60]. As shown in Figure 5b, by transferring the obtained 3D graphene films (3D-GFs) to a flexible PDMS substrate, a reversible change in resistance under large strain or bending was achieved by Pan et al. [49]. The highly sensitized piezoresistive strain sensor can produce a fast response to finger bending. Polat et al. [50] used graphene sensitized by semiconductor quantum dots (GQDs) to construct a photosensitive wearable sensor for the material and a number of prototypes are presented for monitoring key physiological characteristics, including heart rate, arterial oxygen saturation (SpO_2_), and respiratory rate, as shown in Figure 5c.

Reduced graphene oxide (rGO) has been widely used in the fabrication of flexible sensors due to its excellent electrical conductivity, good mechanical properties, and ease of processing. [61,62,63,64]. Du et al. [65] uniformly coated the reduced graphene oxide on the surface of nonwoven fabric (NWF)to prepare a graphene NWF (GNWF) flexible sensor, which has good reproducibility in response to stretching, bending and compression, can respond to a series of human movements of different degrees, and can monitor finger, wrist, and other parts of the pulse, breathing, and other small movements. By Lu et al. [51], rGO and PDMS were deposited on the substrate to form the highly conductive piezoresistive pressure sensor after thermal reduction treatment. The assembled pressure sensors had excellent sensing characteristics and successfully detected various physiological activities and subtle physiological signals, including walking, running, elbow bending, finger bending, breathing, speaking, and blood pulse, as shown in Figure 5d. By mixing tissue paper with GO solution, and converting the GO sample into reduced graphene oxide (rGO) paper by the thermal reduction method, a graphene paper pressure sensor with excellent performance in the range of 0–20 kPa was obtained by Tao et al. [52], which can be applied in pulse detection, breathing detection, voice recognition, and various intense motion detection. As shown in Figure 5e, a dry and self-adhesive strain sensor consisting of a sensing layer and an adhesive layer has been fabricated by Wang et al. [53]. The sensing layer is made of nonadhesive water-dispersible polyurethane (WPU) composites of rGO and CNTs. The adhesive strain sensors are used to monitor body movements with large or small strains, including movements of the fingers, wrists, knees, ankles, and muscles, as shown in Figure 5f.

Compared to carbon-based materials, metal nanomaterials have unmatched electrical conductivity. In 2019, Li et al. [66] fabricated a pressure sensor based on micro-nanowires densely stacked with gold nanoparticles using the imprinting method. The entire assembly process takes only 1 min; the optimal detection limit of the pressure sensor is as low as 25 Pa and it can be applied to any part of the human body. Its high sensitivity ensures its application in real-time monitoring of daily human motion and as an electronic skin for prosthetics. Kim et al. [54] demonstrated a flexible and transparent sensor fabricated by maskless laser processing of Ag nanodendrites and spray coating of Ag nanowires, as shown in Figure 5g, capable of sensing both pressure and position. Zhao et al. [67] combined the natural viscoelastic material of thermoplastic polyurethane (TPU) nanofibers with the conductive material of silver nanowires (AgNWs) to fabricate a capacitive pressure sensor with the characteristics of high sensitivity, fast response time, and low detection limit. Choi et al. [55] used a selective patterning process to embed stretchable and transparent silver nanowire/reduced graphene oxide (AgNWs/rGO) electrode wires into a polyurethane (PU) dielectric layer on the PDMS substrate. A transparent stretchable capacitive touch sensor is fabricated, as shown in Figure 5h. The stretchless touch-sensing function of this sensor has great potential in wearable electronic devices and human–machine interfaces.

Organic polymer materials, another conductive material for flexible tensile sensors after carbon nanomaterials and metal nanomaterials, have gradually become one of the best raw materials for the fabrication of components in the field of sign language gesture recognition due to their unique properties. A flexible piezoelectric haptic sensor array based on polyvinylidene fluoride (PVDF) film was proposed by Yu et al. [68] to measure the triaxial dynamic contact force distribution. The array consists of six haptic units. In each unit, a PVDF film is sandwiched between four square top electrodes and one square bottom electrode, forming four piezoelectric capacitors to measure pressure changes. Due to their excellent flexibility, the sensor arrays can be easily integrated into curved surfaces, such as robotic and prosthetic hands. Rahimi et al. [56] presented a highly stretchable, flexible piezoresistive strain sensor by transferring and embedding the carbonized pattern produced by laser-carbonized polyimide into an elastomeric substrate (such as PDMS or Ecoflex), whose performance far exceeds that of many other previously reported piezoresistive conductive composites and conductive particle films. It can be attached to a latex glove to monitor the finger flexion angle in real time for sign language gesture recognition, as shown in Figure 5i. Zhao et al. [69] reported the use of stretchable optical waveguides for strain sensing in a prosthetic hand. The photonic strain sensors were integrated into a fiber-reinforced soft prosthetic hand and various active sensing experiments were performed to feel the shape and softness of three tomatoes and select the ripe one. To solve the problem of poor flexibility and stretchability of electronic skin, Wang et al. [70] fabricated stretchable transistor arrays using polymer semiconductors. The tactile sensor they developed has extremely high sensitivity and excellent stretchability and can accurately detect the position of small artificial ladybugs, showing a high degree of stability even under pressure and deformation.

### 2.2. Processing Technologies for Flexible Components

There are many ways to prepare flexible sensors, such as dipping and coating, lithography, inkjet printing, screen printing, 3D printing, spinning, thermal drawing, etc. The characteristics of the different processing methods are shown in Table 2. The following describes the most common traditional preparation processes used in flexible sensors integrated into data gloves or hands to sense pressure, strain, and EMG signals.

#### 2.2.1. Dipping and Coating

Dipping and coating are typically used when the base material is fabric. In order to impart sensitive or conductive properties to the fabric, conductive composite materials are often combined with the fabric by dipping or coating and other processes to form fabric sensors that can accurately sense external pressure or tension and are usually used as flexible sensors to measure the movement of the human hand or elbow joint.

**Figure 6:**
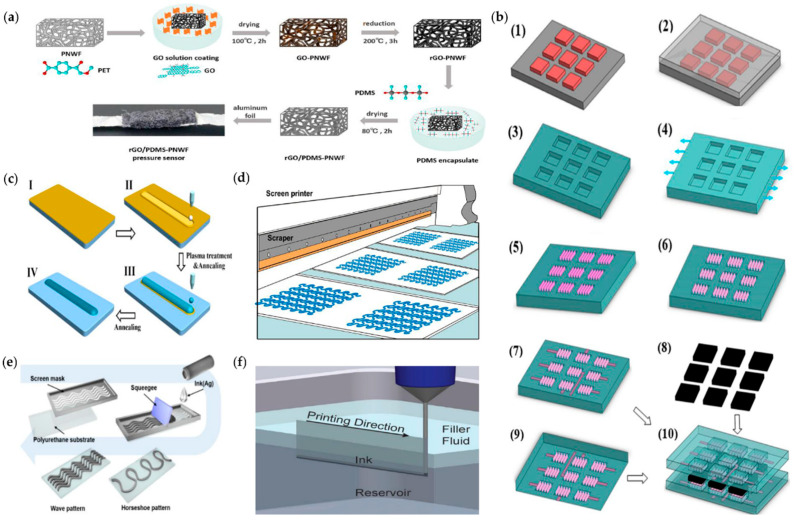
Different process methods for the preparation of flexible sensors for the detection of finger movements. (**a**) Dipping and coating, reprinted from [51], copyright (2018), with permission from Elsevier. (**b**) Lithography, (1) SU-8 mold; (2) PDMS casting; (3) PDMS mold; (4) Pre-strain PDMS; (5) Pre-strain relax after O_2_ plasma treatment; (6) Sodium dodecyl sulfate (SDS) surface functionali-zation; (7) Ag wrinkled electrodes on the PDMS substrate after Ag sputtering; (8) CNTs/PDMS elastomer dielectric layer; (9) Ag wrinkled electrodes on the PDMS substrate; (10) Flexible pressure sensor, reprinted from [17], copyright (2021), with permission from PubMed Central. (**c**) Inkjet printing, (I) Ultrathin Cytop layer deposited on substrate by spin coating; (II) Printing pure solvent to etch the Cytop layer; (III) Printing oxide precursor into surface-energy pattern; (IV) Formation of oxide film after annealing, reprinted from [72], copyright (2017), with permission from American Chemical Society. (**d**) Screen printing, reprinted from [73], copyright (2019), with permission from American Chemical Society. (**e**) Screen printing, reprinted from [74], copyright (2019), with permission from Elsevier. (**f**) 3D printing, reprinted from [75], copyright (2014), with permission from Wiley.

The dipping or coating process has been used in some of the previous literature. As shown in Figure 6a, Lu et al. [51] first dipped the polyester nonwoven fabric (PNWF) substrate in a GO solution, then reduced the coated GO–PNWF, and bonded the aluminum foil to the rGO–PNWF composite as an electrode. The rGO–PDMS–PNWF pressure sensor was prepared by combining the mixture of PDMS resin and hardener with the composite material, which can detect various physiological activities and subtle physiological signals. Ge et al. [71] provide an electronic fabric based on intertwined sensor electrodes, which has the ability to simultaneously map and quantify the mechanical stresses induced by compression, lateral strain, and flexion. This stretchable electronic fabric, with multiple force-mapping properties and high durability, has potential applications in wearable artificial skin for humanoid robotics, biomedical prostheses, and physiological analysis devices.

#### 2.2.2. Lithography

Lithography is a commonly used traditional fabrication process that is relatively complex, costly, and suitable for mass production. The minimum size of graphics produced by lithography can be as small as nanometers, which is suitable for application scenarios that require very high accuracy [76]. The principle of lithography is to use a photoresist to transfer the pattern from the mask plate to the silicon wafer or other media layer and to obtain a specific pattern shape after exposure and development [77]. Lithography processes can use different photoresists (also known as resists) and there are two different processes, namely positive resist lithography and negative resist lithography [78]. As shown in Figure 6b, Cui et al. [17] reported a new type of capacitive flexible pressure sensor, which has the characteristics of high sensitivity, fast mechanical response, wide working pressure range, durability, and good repeatability, etc. The whole preparation process is simple and easy to operate and the resulting strain sensor can effectively detect the position and distribution of finger pressure. The method is found to be compatible with conventional nanomanufacturing technology, which can save costs in practical applications for large-scale production. Bae et al. [79] fabricated a graphene strain sensor by active ion etching, and embossing on elastic plastic, or stretchable rubber substrate. The graphene film was patterned by lithography. The wreath structure enabled the sensor to monitor complex motion or deformation of body parts.

#### 2.2.3. Inkjet Printing

Inkjet printing is usually controlled by a computer program. Compared with traditional fabrication technology, inkjet printing technology has gradually shown its great potential in the field of large-size, high-density flexible electronics due to its advantages of low cost, environmental friendliness, wide substrate applicability, high degree of graphic freedom, high precision, and noncontact [80]. Whether the performance of the flexible sensor of inkjet printing is excellent depends on whether the stability of the conductive ink of inkjet printing is good. At present, inkjet printing conductive inks mainly include transparent oxide ink, carbon ink, and metal ink, the advantages and disadvantages of which are listed in Table 3. As with the flexible sensor manufacturing materials, the difference in inkjet printing materials will make the stability, sensitivity, and stretchability of the sensor different, so there is a difference in the perception of human hand movement.

#### 2.2.4. Screen Printing

Screen printing is performed by tilting a squeegee to deposit ink on a screen in a specific pattern on the substrate [91]. In recent years, screen printing electronics technology has developed rapidly, mainly because inkjet printing generally requires the conductive material in the ink to be less than 100 nm. Screen printing ink requirements are not as stringent as inkjet printing, making screen printing more conducive to the fabrication of large-area sensor arrays. To overcome the limitations of elastic electronics, such as cost, toxicity, or inability to pattern on a wide range of substrates, Tang et al. [73] developed an ink consisting of liquid metal particles and desirable polymer solutions for screen printing, which can be tuned to print on different surfaces and avoid toxic organic solvents in most cases, as shown in Figure 6c. Yoon et al. [74] used screen printing to fabricate a low-cost and stretchable Ag nanoparticle (NP) electrode on polyurethane (PU), as shown in Figure 6d. The resulting strain sensor has high stretchability, shows a stable response in the 20% strain range, and can be applied to the skin of human hands to measure changes in hand motion resistance. Liu et al. [92] designed a printable nanocomposite with a pearl-inspired hierarchical structure, diffusible components, and rich dynamic interactions for the construction of a healable and durable strain sensor. This strain sensor was fabricated by a screen-printing technique with the printing force, speed, and angle between the squeegee and stencil specifically optimized for the GO-AgNW-based inks, which can repeatedly and effectively self-heal with simple water treatment, greatly extending its lifetime and cycle life (over 10,000 cycles). Tian et al. [93] prepared Ag nanodendrite (ND) inks with good printability for a variety of substrates, which can be directly screen-printed onto nitrile rubber to fabricate strain sensors. Their different strain ranges and sensitivities can be obtained simultaneously by printing versatile geometric patterns. Finally, a smart glove based on PSSs is used to monitor human movements (finger bending, wrist bending, walking, etc.) and gesture actions.

#### 2.2.5. 3D Printing

In today’s rapidly changing science and technology, it is difficult for traditional processes to process flexible sensors with complex functional structures, which greatly restricts the function of flexible sensors; so, 3D printing technology has gradually attracted people’s attention. 3D printing technology is a new type of 3D functional device fabrication technology, which can fabricate flexible devices with complex geometric shapes through layer-by-layer assembly based on 3D digital models [94]. Based on the classification of materials, 3D printing can be divided into five aspects: 3D printed molds, the flexible sensor substrate and sensor body, the sensing element, the flexible and stretchable electrodes, and fully 3D printed tactile sensors [95]. Christ et al. [96] used dual nozzle 3D printing technology to fabricate uniaxial and biaxial strain sensors with conductive pattern designs that could be incorporated into wearable gloves to measure finger curvature. Such sensors have potential applications in wearable electronics, soft robotics, and prosthetics. Yin et al. [97] constructed an ionic conductive hydrogel using the 3D printing technology of photopolymerization. Based on the transparent and highly elastic hydrogel, a capacitive sensor was developed that could sense pressure and strain and determine skin position by collecting body signals. Muth et al. [75] reported an embedded 3D printing method, shown in Figure 6f, where conductive ink is extruded directly through a deposition nozzle into an elastic reservoir, where the ink forms a resistive sensing element and the reservoir serves as a substrate material, creating a highly stretchable strain sensor that can be embedded in a data glove to measure gesture changes. Leigh et al. [98] presented the formulation of a simple conductive thermoplastic composite called “carbomorph” and its use in a low-cost 3D printer to print electronic sensors capable of sensing mechanical bending and capacitance changes.

#### 2.2.6. Spinning

Spin coating is a method of obtaining a small film size by using centrifugal force to diffuse the material and distribute it uniformly on a flat substrate. For some specific materials, the thickness of the film is mainly determined by the concentration of the material, the viscosity, the volatility of the solvent, and the rotational speed of the spin coater [99]. Spinning is typically followed by an annealing process that evaporates residual solvents, sinters nanoparticles/nanowires, and transforms the spin-coated film into a final transparent electrode. Spin coating is typically followed by an annealing process that evaporates residual solvent, sinters nanoparticles/nanowires, and then transforms the spin-coated film into the final transparent electrode. Moschogiannaki et al. [100] mixed CoV_2_O_6_ nanoparticles (40 mg), ethyl cellulose (Fluka, 30–70 mPa·s), and a-terpinol (Aldrich, 90%) and spun the uniformly ground solution onto the glass substrate. The film to equip the finger-fork electrode was obtained by spinning the coating at 700 rpm for 10 s and then at 3000 rpm for 30 s. The surface morphology and film thickness of the sensor material were observed by scanning electron microscope, and it was concluded that the spin-coated sensor on the glass substrate had a higher response at room temperature. The response and recovery times were 94 s and 74 s, respectively. In addition, traditional spin-coating techniques are constantly being optimized to create high-performance devices. For example, Yuan et al. [101] proposed an “eccentric” spin-coating method in which the center of the substrate is moved 20–40 mm away from the rotation axis of the spin-coating machine. The centrifugal force in this improved technique is different from that in center spin coating. The solution is diffused radially outward to promote the unidirectional orientation of the prepared off-center spin-coating (OCSC) films. However, although the spinning process is very low cost, it has no chance of being used in mass production because of the solution loss caused by the spin coating process. 

#### 2.2.7. Thermal Drawing

Thermal drawing (TD) is a process that thermally stretches fibers of complex geometry and multiple materials to a size of 100 microns [102]. Liu et al. [103] proposed a flexible optical fiber temperature sensor made by thermal stretching of a variety of materials, which demonstrated sufficient sensitivity over a temperature range of 0–285 °C with a fast response and recovery time of 11.6 and 14.8 s, respectively, in addition to being sewn onto everyday fabrics and gloves. It has a highly stable performance in response to body temperature changes and touch temperature detection. The composite material can obtain excellent melting point and fluidity through the hot drawing process but it needs to be clad with some special materials to achieve this. For example, Lee et al. [104] prepared and fabricated macro preforms of poly (vinylidene fluoride-trifluoroethylene) (VDF-TrFE) and carbon black (CB)–polypropylene (PP) composites by thermal drawing process to fabricate highly flexible piezoelectric fibers. They proposed a polypropylene polymer with high-yield stress. The amorphous poly (vinylidene fluoride-trifluoroethylene) (P(VDF-HFP)) polymer is used as the coating material for this piezoelectric fiber. Since the melting point of this material is 120 °C, in order to overcome the incompatibility between the polymer and its internal composite at tensile temperature, the material geometry and TD parameters were optimized to produce fibers with a length of more than 80 m. After annealing and polarization, the resulting fiber showed extremely excellent strain characteristics. However, the interfaces between different fiber components obtained by thermal stretching are thermodynamically unstable due to their different surface tensions [105,106], which requires further processing of these fibers by some methods (e.g., laser and heat treatment), which can produce another type of microstructured multimaterial fibers [107].

## 3. Application of Flexible Sensor Technology to Manipulator Control

The continuous development of various flexible substrate materials, sensor materials, and conductive materials has laid the foundation for the production of flexible sensors with various functions. With the continuous progress of preparation technology, the application fields of flexible sensors are expanding. Based on the research on the manufacturing materials and technology of flexible sensors, this paper introduces the application of flexible sensors in the field of manipulator control.

The development of robots requires the combination of knowledge and technology from various fields, such as manufacturing, sensor technology, and artificial intelligence. The requirements of the robot for high intelligence, good flexibility, and strong versatility are constantly being realized. The manipulator can respond to changes in hand posture using data-glove control or surface EMG control based on flexible sensors. The data-glove control method usually takes the flexible sensor element as the core component, which is designed as an intelligent electronic device integrated into the data glove for robot hand control. The surface electromyographic signal is used to control the manipulator through pattern recognition of the collected information.

### 3.1. Flexible Sensor Integrated into Data Glove for Manipulator Control

Researchers often use flexible sensors integrated into data gloves to capture changes in gesture state, which are then transmitted to the manipulator via an external connected device for gesture simulation. The first glove-based systems were developed in the 1970s and a number of different designs have been proposed since then. Early glove prototypes included the Sayre Glove, the Massachusetts Institute of Technology (MIT)-LED glove, and the Digital Entry Data Glove [108]. Beginning in 1987, American scientists began to apply flexible printing technology to data-glove research and it slowly became popular. The first commercially available data glove appeared in 1987, an improved version of the first DataGlove developed by Zimmerman in 1982 [109,110]. The technology was similar to that used in the Sayre Glove in 1977. In 1990, Eglowstein [111] reported on three commercial hand trackers: VPL Research’s DataGlove, Exos’ Dexterous Hand Master, and Mattel’s Power Glove. In 1999, LaViola [112] conducted a survey of hand posture and gesture-recognition techniques and technologies.

**Figure 7:**
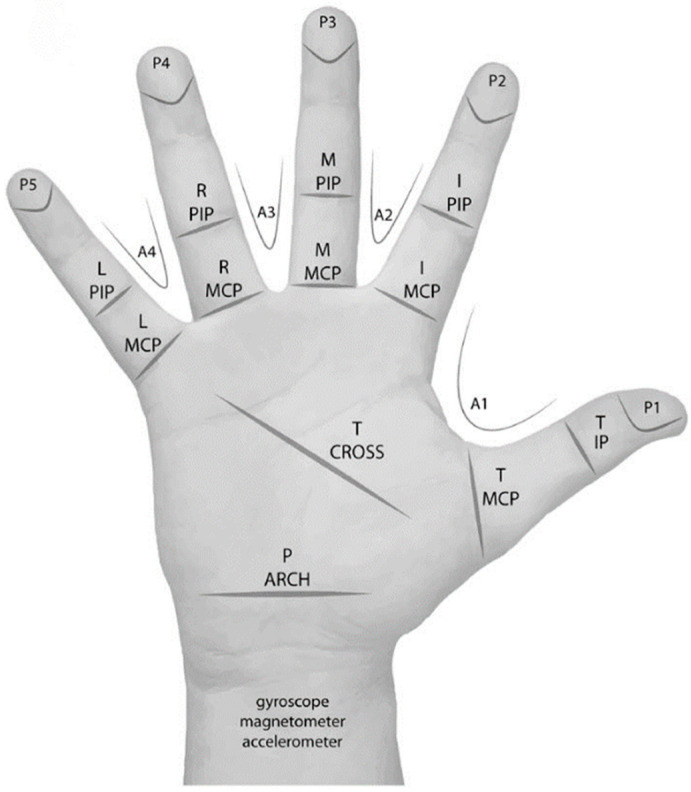
Labels and sensor locations of the VMG 30™ data glove proposed by Krammer et al. consisting of 29 sensors reprinted from [113], copyright (2020), with permission from PubMed Central.

In general, the position of the flexible sensor on the data glove is divided into three parts: (1) the installation of the finger joint to measure the amount of movement in the metacarpophalangeal (MCP) and proximal interphalangeal (PIP) joints as the gesture changes; (2) the installation on the fingertips to measure pressure; and (3) the carpal movements were detected in each part of the palm, as shown in Figure 7 [113]. The number of sensors is related to the degree of freedom to be measured and, in general, a position requires a flexible sensor.

The flexible sensor integrated into the data glove mainly has two types, piezoresistive and fiber optic, according to the different sensing principles. The flexible piezoresistive sensor is a type of flexible pressure sensor, which can convert the external pressure or strain stimulus signal into an electrical signal by the piezoresistive mechanism. There are also two types of flexible strain sensors: resistive and capacitive. The resistive sensor converts the external signal (pressure, strain, etc.) into a change in resistance for detection. The capacitive sensor converts the measured pressure into a change in capacitance value as a sensitive element. The fiber optic sensor is based on the photoelectric principle, which converts the optical signal into an electrical signal that can be measured and recorded to achieve detection. Table 4 shows the advantages and disadvantages of the various flexible sensors.

For different sensor technologies, the choice of the right material for the research is also important. Clauser et al. [114] proposed a stretch-sensing soft glove composite of silicone and textile layers to interactively capture hand poses with high accuracy and without the need for an external optical setup. Sundaram et al. [115] designed a scalable tactile glove that, in combination with deep convolutional neural networks, represents the sensors distributed over the hand and can be used to identify individual objects, estimate their weight, and explore the typical tactile patterns that occur when objects are grasped. Based on the poly(acrylamide) (PAAm) hydrogel, a strain sensor with high stretchability and sensitivity was designed and fabricated by Hang et al. [116]. Then, a smart glove was fabricated by the coupling of multiple strain sensors and the corresponding circuit. The smart glove is capable of expressing and recognizing American Sign Language and can be used to wirelessly control a robotic hand through hand gestures. A biologically inspired soft robotic thumb rehabilitation system has been developed by Maeder-Yorkto et al. [117], which is capable of reproducing the motion path of a thumb during opposition grasping. The integration of this with a lightweight hand fixation and a compact control system resulted in a promising prototype for a wearable, home-based, task-oriented thumb rehabilitation device. A multisensor glove controller was designed by Jhang et al. [118] to control a mobile robot and a six-axis robotic arm for industrial operations. The user can monitor the situation in front of the mobile robot arm and record the trajectory and position coordinates, thus achieving the functions of remote control. Pu et al. [119] developed a triboelectric quantization sensor for joint motion and constructed a synchronous control system for a manipulator capable of grasping objects. Using the sensor on the data glove to measure the bending or opening angle of the finger, and then finding the mapping relationship between the sensor measurement data and the change in hand posture, the researchers continue to work on finding this more accurate mapping relationship. A more detailed summary of the sensor technology used in data gloves is shown in Table 5.

There are many ways to integrate flexible sensors into data gloves. The most common attachment method is fabric filling [134,136,137], in which strain and fiber optic sensors are placed between multiple layers of fabric in fabric gloves. This method is easy to use, but the gloves can become bulky and interfere with gesture changes. Some data gloves made of flexible materials, such as silicone gloves, can use the method of printing ink directly onto the glove [138], which solves the weight problem of the glove, but the ink may volatilize or degrade under the influence of air, affecting the accuracy of gesture recognition. Of course, conductive yarns can also be used to directly weave knitted sensor data gloves [139] and there are many studies on the performance of such knitted sensors and their potential for gesture recognition. In addition, depending on the material of the flexible sensor and the structure of the glove, methods such as gluing [140,141], stitching [142], carabiner [143], and tape [144] can be used. At the same time, when installing the sensor, it is necessary to secure the sensor to the outer surface of the glove to ensure that there is no relative movement between the sensor and the glove. This can make the data glove more accurate for measuring finger curvature [134]. During installation, it is also necessary to determine the stress distribution on the glove and determine the best location for installation to extend the life of the data glove without compromising dexterity [141]. It is also necessary to eliminate the interaction between sensors in different positions when the number of integrated sensors on the data glove is large. In [140], a linear regression model is used to improve this integration.

**Figure 8:**
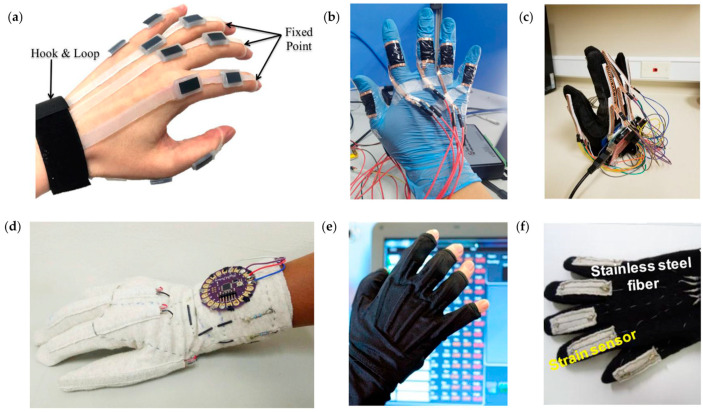
The images of gesture recognition manipulators based on different sensor technologies. (**a**) reprinted from [151], copyright (2016), with permission from Spring Nature. (**b**) reprinted from [144], copyright (2023), with permission from Elsevier. (**c**) reprinted from [147], copyright (2021), with permission from Elsevier. (**d**) reprinted from [152], copyright (2019), with permission from Sensors. (**e**) reprinted from [132], copyright (2022), with permission from Sensors. (**f**) reprinted from [153], copyright (2017), with permission from American Chemical Society.

As integration continues to improve, more and more research has been conducted on robots that receive the motion information collected from human hands and mimic human hands to perform the corresponding action with increasing accuracy. Nassour et al. [140] proposed a versatile soft-sensing glove using commercially available silicone tubing to house the conductive fluid. Fourteen sensors were attached to the glove to measure flexion–extension and abduction–adduction, successfully replicating hand movements. The machine-learning algorithms were used to estimate the angles of the joints in the hand and also to identify 15 gestures, with a classification accuracy of 0.885. Pan et al. [145] presented a wireless smart glove based on multichannel capacitive pressure sensors that can detect 10 American Sign Language gestures at the edge of the glove. In this system, 16 capacitive sensors are fabricated on a glove to capture the hand gestures. The highest test classification accuracy achieved by our system is 99.7%. Maitre et al. [146,147] proposed a new prototype of a data glove that is simple, cheap, reproducible, and efficient (∼100% correct predictions) for object recognition by abstracting the entire theory of gesture recognition. In real life, such a device could be very useful to monitor the evolution of hand dysfunction in Alzheimer’s disease. To solve the problem of separating meaningful dynamic gestures, Lee et al. [148] proposed a gesture-spotting algorithm based on deep learning that detects the beginning and end of a gesture sequence in a continuous data stream. The three algorithms (gesture spotting, sequence simplification, and gesture recognition) were unified into a real-time gesture-recognition system and tested with 11 dynamic finger gestures in real time. Ayodele et al. [149] proposed a piezoresistive data glove using convolutional neural networks (CNN) on six capture classification scenarios. Based on the CNN algorithm, the average classification accuracy was 88.27% and 75.73% for visible and invisible objects, respectively. By sewing reduced graphene oxide (RGO)-coated fibers onto a textile glove, Huang et al. [150] fabricated a flexible and low-cost data glove that was used to monitor the movement of ten finger joints of a hand. Experimental results show the good stability and repeatability of the data glove, with recognition accuracies of 98.5 and 98.3% in different test scenarios. Figure 8 shows the images of gesture recognition manipulators based on different sensor technologies.

### 3.2. Manipulator Control Application Based on Surface EMG Signal

The flexible sensor has excellent performance, is convenient and flexible, and can be made into flexible electronic devices to obtain rich and diverse signals from the human body by wearing on the hand as a command source to control the robot hand. Among them, surface electromyography (EMG) is a noninvasive method of recording EMG signals. The EMG signals from the muscles of the forearm can be used to detect hand grips and gestures. The surface EMG signal is relatively easy to record. With the development of sensor technology, the EMG signal of the forearm of the human body is collected by flexible wearable sensors to identify the posture change of the hand and to realize the following or synchronous posture change of the manipulator and the hand.

Siomau et al. [154] processed the electrical nerve signals collected from the surface of the residual limb muscles and used them to control the prosthesis for different movements. By assuming that there are distinguishable and repeatable signal patterns between different types of muscle activation, the prosthesis-control problem was reduced to a pattern-recognition problem and verified. Lopes et al. [155] created a soft, ultra-thin, stretchable electronic skin by printing patterns on temporary tattoo paper using a desktop laser printer and then coating it with silver ink and a eutectic gallium indium (EGaIn) liquid metal alloy that self-adheres to the human epidermis to collect EMG signals to control robotic prostheses. Huang et al. [156] created a scalable human–machine interface test platform based on a four-layer design that provided eight-channel sensing, collected acceleration, angular velocity, and surface EMG signals, and controlled the translational rotation and grasp of the robotic arm, respectively, via the platform’s Bluetooth data communication function. Leigh et al. [157] propose a wearable machine–joint interface device that enhances our innate capabilities by providing additional machine joints, enabling “collaborative interaction” where the movement of the machine joints can be controlled through an interface with our muscle signals as a direct extension of our body.

**Figure 9:**
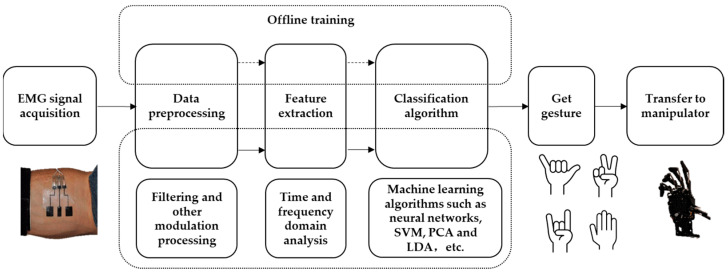
EMG detection process.

The movement intention issued by the brain triggers the excitation of the motor cortex of the brain, which sends movement instructions to the α motor neurons of the spinal cord and, then, transmits the relevant movement information to the muscles of the human forearm, resulting in the release of calcium ions in the muscles, causing the muscle to contract to produce a biological current, which reaches the surface of the skin through the tissue fluid and sebum, showing a current difference between the surface electrodes [158]. The EMG signal is collected by measuring the current difference through the electrode. Then, the signal is preprocessed and the feature is extracted to realize the functions of filtering and noise reduction. Finally, the data is trained and the pattern is classified. The classification results are sent to the manipulator, which performs the appropriate action by controlling the toggle drive motor. Like the human hand, the manipulator has five fingers, each finger is independently driven by a motor, and each finger joint is connected by a toggle mechanism. Upon receiving the signal, the controller drives the motor to rotate, which simultaneously drives the middle and the end joints to rotate, allowing the mechanical finger to bend and extend along a fixed path. The identification process is shown in Figure 9.

First, the surface EMG signal is recorded on the skin using a surface electrode. When applying the electrode, the skin should first be wiped with alcohol to ensure good contact between the electrode and the skin. Second, the sampling time should not be too long each time and the electrode should be removed after sampling to avoid allergic reactions to the skin caused by contact with the electrode. Then, the arrangement of the electrodes directly affects the strength of the EMG signal. Currently, there are three different arrangements: (1) according to the experimental requirements, after the target muscle to be sampled is fixed, the electrodes are applied in pairs [159]; (2) the electrode is evenly distributed on the skin surface in a ring structure according to a certain law; and (3) the electrodes are closely arranged to collect the EMG information in all directions [160]. In addition, studies have shown that a stronger EMG signal can be obtained by placing the sensing electrode in the muscle belly and a weaker EMG signal can be obtained by placing the electrode at the edge of the tendon or muscle group [161].

The surface EMG signal is very weak and has poor stability, so it is necessary to pre-process the signal. This step helps to improve the quality of the signal, which can be mainly divided into two steps: signal segmentation and signal filtering [162]. In the process of signal acquisition, the signals of different channels are superimposed on each other, which will cause the characteristics of the signals between channels to be similar, resulting in identification errors, and this interference is called signal crosstalk [163]. Signal segmentation can effectively solve the crosstalk problem, help to extract features, and reduce the dimension of data. The specific method is to use windowing technology to define the size and step size of the EMG waveform during data acquisition. The input data points are defined as the window size and the step size is the different data points between two consecutive windows. Kunapipat et al. [164] built a support vector machine (SVM) model to classify gestures. When the overlap window size was 100 samples, the overlap was 50% and the maximum average classification accuracy was 91.28%. Similarly, Tepe et al. [165] used the windowing technique in the data preprocessing stage and confirmed that the subspace K nearest neighborhood (SKNN) method achieved a classification success rate of 95.8% in the 100 ms 50% overlap window. Wen et al. [166] investigated the influence of the window size and step size of the input EMG signal on the sensitivity and accuracy and finally concluded that the optimal window size and step size of the multi-input deep convolutional neural network were 120 and 20 data points, respectively. Chen et al. [167] set the sliding window length to 150 sample points (73.2 ms) in the preprocessing of EMG signal data and the accuracy rate of gesture recognition exceeded 98%. Signal filtering is to eliminate interference and noise reduction The specific operation is, first through the signal conditioning circuit, using the difference of the signal collected from different channels as the signal input to filter out the common mode interference, and then through the filter to remove the noise outside 10~500 Hz [168]. For example, Li et al. [169] used a 50 Hz comb filter to eliminate power frequency interference, which is the interference signal generated by alternating current in electrical equipment through electromagnetic radiation and is the main noise in the whole frequency range of EMG. In addition, the algorithm based on wavelet transform is also used for signal filtering, because it is suitable for various signals. Wavelet decomposition is to decompose the EMG and, then, set a certain threshold to quantify a certain frequency; and the waveform after the threshold quantization is basically the useful EMG signal in the original signal [170,171].

In order to control the manipulator by surface EMG, the most important thing is to extract and classify the EMG feature. The EMG feature extraction methods usually require time domain analysis, frequency domain analysis, and time–frequency domain combination analysis. The time domain features mainly include mean absolute value, root mean square, and so on. The frequency domain features include power spectral density, mean frequency, median frequency, and autoregressive coefficient. Time–frequency domain analysis mainly includes short-time transformation, Winger–Ville transformation, and so on. The classification methods of the EMG signal mainly include neural network, fuzzy algorithm, probability estimation-based algorithm, support vector machine (SVM), principal component analysis (PCA), linear discriminant analysis (LDA), and so on.

More specifically, an automatic recognition algorithm for identifying hand movements from surface EMG signals has been proposed by Fatimah et al. [172]. Two publicly available datasets are used to test the effectiveness of the proposed algorithm. With an average accuracy of 99.49% on the UCI dataset and 93.53% on the NinaPro DB5, the proposed method outperforms the state-of-the-art algorithms. The differences between the electromyography (EMG) patterns of normal subjects and amputees were investigated by Campbell et al. [173]. Using previously collected EMG data for different wrist, finger, and grip potentials of 20 able-bodied subjects and 10 amputees, the results of unsupervised cluster analysis show that a simple linear classifier can discriminate able-bodied and amputee subjects with 90% accuracy using multiple gesture EMG. Four machine-learning (ML) algorithms, support vector machine (SVM), random forest (RF), bagged tree, and extreme gradient boosting (XGBoost) were used by Alam et al. [174] to classify hand gestures using an electromyography (EMG) dataset; the prediction accuracy of these algorithms was compared with long short-term memory (LSTM). XGBoost provided the highest accuracy, of approximately 97%, while LSTM provided a superior accuracy of nearly 99%, which promises to provide physiologically natural upper limb movement control. The reason why LSTM provides higher accuracy is because LSTM achieves better classification by learning more parameters and selectively remembering them over a long period of time. Lucas et al. [175] proposed a supervised classification method for multichannel surface EMG signals, using a support vector machine (SVM) to classify them in the multichannel representation space, and applied the method to the classification of six hand movements. The mean misclassification rate (mean ± S.D.) for the classification of eight channels in six subjects was 4.7 ± 3.7%. Alkan et al. [176] used discriminant analysis and a support vector machine (SVM) classifier to classify recorded EMG signals generated by the biceps and triceps muscles for four different movements. The SVM classifier gives a very good average accuracy rate (99%) for four movements, which can be used to classify EMG signals for prospective arm prosthesis-control studies. A homemade four-channel sEMG amplifier circuit was designed by Baspinar et al. [177] to measure sEMG signals. Seven different movements were classified and their classification performances were compared. The classification rates of artificial neural network (ANN) and Gaussian mixture model (GMM) classifiers were compared. For other EMG acquisition devices, their classification methods, and recognition accuracies are listed in Table 6.

## 4. Existing Issues and Solutions

This paper reviews the research on manipulator control based on flexible sensor technology and summarizes the existing problems in this field from three aspects: materials, processing technology, manipulator-control system design, and the corresponding solutions.

### 4.1. Problems and Solutions Related to Materials

Flexible sensor materials cover almost all categories of organic or inorganic materials, including liquids, gels, and solids. In order for flexible sensors to be better embedded in data gloves or to fit on the arm, flexible materials must have improved mechanical properties, biocompatibility, and electrical conductivity. Researchers aim to achieve this by preparing composite materials, microstructuring substrate materials, or modifying the structure of existing materials.

(1) Preparation of the composite materials: the core component of the flexible sensor is the composite material formed by mixing the conductive sensing material with the base material, which has a strong relationship with the sensitivity, linear range, and response time of the sensor. For example, it is difficult for simple carbon-based materials to form an ordered arrangement at the macro level, and a disordered arrangement will weaken the conductivity of the material itself. The use of composite materials is an effective way to maintain good conductivity in flexible sensors under high strain. Traditional electrode materials cannot meet the demand for flexibility, so researchers can effectively solve this problem by using composite materials as electrodes and developing stretchable capacitive sensors. If the composite material is not strong enough, it may affect the stability of the flexible sensor and special processing methods are usually used to improve the stability of the sensor;

(2) The problem of limited substrate elasticity is solved by microstructuring: The substrate material not only affects the elastic deformation performance of the sensor but also has a critical effect on the sensing performance. The microstructure of the substrate material is one of the important methods to improve the performance of the sensor. The microstructured substrate film can not only improve the elastic deformation performance of the sensor but also make the flexible sensor have higher sensitivity and faster response time than the unstructured flexible substrate film. The traditional microstructure is mainly obtained by a lithography process. The micropattern mold is first prepared by the manufacturing process. Then, the required solvent is spin-coated on the mold after stirring and degassing. Finally, the microstructured base material is stripped after curing. In addition, by changing the structure of the existing material, the flexible sensor can have better mechanical properties. In the case of nonstretchable rigid materials, special processes can also be used to make them flexible and able to withstand a certain amount of strain.

### 4.2. Problems and Solutions in the Preparation Process

The choice of printing process is the key to low-cost, rapid, and large-scale production of flexible sensors. At present, various printing processes are endlessly emerging, to a large extent to solve the traditional lithography technology caused by high manufacturing costs, a complex preparation process, not having large-scale production conditions, and other problems. However, the printing process also has some impact on the sensitivity of flexible sensors, mainly in the following aspects:

(1) The materials used in printing are different and changing the ratio of materials can give the sensor different resistivity, resulting in different circuit conductivity. (2) The viscosity of the material has a certain impact on the shape of the sensor circuit; if the ink viscosity is too large, it will lead to poor ink transfer in the printing process and paste plate. If the viscosity is too low, the ink is too thin and it will cause printing or migration and ink infiltration. (3) In some printing resistances, the pattern design of the screen will directly affect the accuracy of printing. (4) In the whole process, there will be a small deviation from the design value, that is the manufacturing tolerance, mainly due to the temperature and humidity in the air, and some uncontrollable factors in the preparation process, so as to reduce the preparation tolerance as much as possible, and then improve the sensitivity.

In summary, the quality of the printing process directly affects the performance of the electronic device, and the preparation process needs to be further optimized.

### 4.3. Manipulator Control System Problems

Both data-glove-based manipulator control and human EMG-based manipulator control have room for improvement in terms of cost, type of information collected, and recognition accuracy.

(1) Cost problem of the data glove: although there are already many data-glove products in the market, and researchers are constantly innovating in this field, data glove will incur a lot of costs during the production process, including the cost of materials required to manufacture the glove itself and the flexible sensor and the cost of external circuit boards or some other hardware production, which makes it difficult to put this device into practical use in large numbers. In the future, the cost of using data gloves will be reduced and their development in the market will be more convenient;
(2) The category bottleneck problem of manual movement information collected by the data glove: the generation of an electromyographic signal is due to the hungry excitation of the cerebral cortex, while the manual movement information collected by the data glove is the pressure or angle change generated when the gesture changes, obviously the electromyographic signal is more abundant;
(3) Improving the sensitivity/accuracy: first of all, the type of material used has an impact on its accuracy. For strain sensors, the better the piezoresistive properties of the material, the better the performance of the sensor; for pressure sensors, the electrical conductivity of the materials greatly affects the accuracy of signal conversion in collecting useful information and, then, affects the accuracy of detection. The photoelectric properties of the material affect the sensing performance of the optical fiber sensor. Secondly, the design of the circuit affects the sensitivity/accuracy. The acquisition system generally uses the computer as the hardware platform and the A/D converted EMG signal is sent to the computer by the data acquisition circuit for postprocessing. The higher the sensitivity of the circuit design, the better the circuit design must be to improve the sensitivity. Finally, the design of the classification algorithm affects the sensitivity/accuracy. In research, it is best to compare and select different classification model algorithms and select the one with the highest accuracy for pattern recognition to achieve better control of the manipulator.

### 4.4. Commercial Application

Although there are still many problems in this area of research, researchers continue to conduct research to promote the solution to the above problems. However, all research should be based on the actual application environment. At present, researchers have conducted product research and development in the application areas of sign language recognition, human–computer interaction, and robot control based on a flexible strain sensor. The summary of the relevant fields is shown in Table 7.

The application of sign language recognition has been very extensive, taking into account the visual, auditory, and language barriers of three cases; the existing technology has been able to use smart devices [190,191,192] to convert sign language into speech or text to facilitate the daily life of deaf people.

Of course, researchers are also studying different languages in different countries or regional dialects and developing devices accordingly [153]. By capturing human gestures and actions to control the robot in real time is also a basic application, which can be derived to the field of rehabilitation medicine and clinical medicine. When machines are used instead of manual rehabilitation training, time and labor are saved as new clinical application products are constantly being designed, and have proven their good application prospects [6,199,200,201]. Among them, the exoskeleton manipulator designed in literature [6] has strong environmental resistance and has been able to grasp 3 kg heavy objects underwater many times without damage. In the literature [200], collecting surface EMG signals from the user’s arm to control the limb for rehabilitation movement can realize independent fine assisted movement with different mechanisms, especially the assistance of a single finger, which proves the possibility of commercial application with experiments. Furthermore, in the literature [204], a picking manipulator was designed using commercially available components, which achieved an average picking time of 5.93 s and a standard deviation of 0.26 s. From a commercial point of view, it can replace manual picking and create huge economic benefits in fruit harvesting. Manipulator control based on flexible strain sensor is also a kind of human–computer interaction. We can establish communication between the robot and the virtual world based on a flexible sensor [193,194,195]. In particular, it can also be used for VR-based surgical training; by recognizing different hand-grasping movements [196], it is possible to determine the type of surgical tool used by the doctor and display it in the VR space. The trainer can then use gestures to control the tools for surgical training.

## 5. Conclusions

This paper summarizes the literature in the field of manipulator control by data glove or by EMG based on flexible sensor technology. Since the flexible sensor can be attached to the data glove or attached to the human arm to collect signals, it has the characteristics of being natural and direct, so it has gradually replaced other methods to become the focus of manipulator control research. The choice of substrate material, sensor material, and electrode material has a great impact on the sensitivity of the designed flexible sensor. It is particularly important to choose the right material, combined with the design of the microstructure and microchannel, to produce a flexible sensor with high sensitivity, extensibility, and good conductivity, which will greatly reduce the problems existing in the process of gesture signal acquisition. The bend sensor integrated into the data glove detects the degree of finger bend and the angle of finger opening, realizes the monitoring of all finger joints, enhances the gesture perception ability, and improves the accuracy of gesture discrimination. By processing the surface EMG signal collected by the strain sensor attached to the human arm to judge the posture of the human hand, the EMG signal contains very rich information, and the online training of the manipulator with the basic algorithm can constantly improve the recognition rate.

In terms of commercialization potential, flexible sensors are promising due to their scalable manufacturing process, excellent performance, and good biocompatibility. Many devices are now commercially available or have been proven in academic laboratories, demonstrating the feasibility of moving from R&D to large-scale commercial products, but there are still many unknown challenges to be solved in the field due to the diversity of device characteristics:

(1) In the manipulator control research, the real-time performance and the accuracy of the manipulator after receiving the signal are equally important. In the future, based on the experimental verification, the real-time performance should be considered from the whole control system, the analysis should be carried out from the active end to the slave end, and the hardware design of the manipulator should be further studied to further improve the real-time performance and accuracy. Simple and effective algorithm design will continue to be a hot spot in the future research of the manipulator and better algorithms can be explored in the future to improve the accuracy of model recognition and ensure the effectiveness of real-time control. (2) With the development of human beings into deeper fields, the control of the manipulator also needs to be transferred from the ordinary end-to-end to the interactive mode; that is, the manipulator receives the signal of the human hand to complete the specified action and gives back to the human hand so that the human can decide whether to change the decision according to the feedback signal. This interaction technology can make the use of the manipulator more practical. On this basis, flexible sensor technology can be applied to other parts of the body to identify human movements projected onto the robot and the robot can be remotely controlled to act according to the user’s intention.

## Figures and Tables

**Table 1:** Comparison of physical properties and advantages/disadvantages of common substrate materials.

Main Substrate Materials	TE	MBP	ROC	DA	YM	Highlight Advantages	Disadvantages
PDMS	+	+	+++	+	-	There is a relatively wide range of application scenarios.	Poor mechanical properties, low surface hardness, easily scratched.
PI	+	+	++	+	++	Excellent mechanical properties.	High transparency, low adsorption to material.
TPU	+++	+	+++	+	++	High strength and elasticity.	High production cost, cannot be used in high-temperature environment.
PET	+	+	+++	+	++	High stability and wear resistance.	Strict processing conditions.
Paper	+	-	+++	+++	+++	Biodegradable and reusable.	Paper print pattern has many limitations.
Textiles	-	+	+	++	+++	Excellent permeability.	Conductive layer is difficult to distribute uniformly on the substrate surface.

‘-’ means bad; ‘+’ means good; ‘++’ means better; ‘+++’ means excellent.

**Table 2:** The advantages and disadvantages of the various processing technologies.

Processing Technologies	Advantage	Disadvantage
Dipping and coating	Simple and easy operation.	Coated conductive layer only covers the surface of the substrate, which has a negative effect on the durability of the material and reduces the comfort of the flexible device.
Lithography	Suitable for mass production, high precision scenarios.	The process is time-consuming, complex, and costly.
Inkjet printing	Can produce high-resolution printing, low material waste, suitable for large area deposition.	High ink requirements, require low viscosity ink, not suitable for large area printing, not suitable for making complex and multilayer electrodes.
Screen printing	Simple setup, easy to prepare large area sensor array.	Screen printing pattern resolution is limited, screen plate is easy to jam, difficult to clean.
3D printing	Ability to create any geometric shape, enabling the manufacture of complex structures, precise size control.	Slow manufacturing process, not suitable for mass production, limited design flexibility.
Spinning	Can form a thin, uniform film on a flat substrate.	Spin coating wastes a lot of raw materials; when performed on large substrates, easy to make uneven due to different centrifugal speed at different positions.
Thermal drawing	High stability, suitable for large scale preparation.	Requires the drawn material to maintain a specific structure at the drawing temperature.

**Table 3:** Comparison of three inkjet print materials.

Type of Material	Main Materials	Advantage	Disadvantage	References
Transparent oxide ink	Tin-doped indium oxide (ITO), Aluminum-doped ZnO(AZO)	Good light transmission and stability	Not as conductive as metal	[72,81,82]
Carbon ink	CNT and GO	Excellent performance, very low sintering temperature, low material cost	Poor thermal stability and complicated process	[83,84,85]
Metallic ink	Gold, silver, copper, etc.	Comprehensive performance is the best	Relatively expensive	[86,87,88,89,90]

**Table 4:** Comparison of different flexible sensors.

Type of Flexible Sensor		Advantage	Disadvantage
Flexible strain/pressure sensor	Resistive	Sensing mechanism is simple, easy to extract the signal.	Poor repeatability, high hysteresis, high power consumption.
	Capacitive	Operating principle is simple and clear.	Susceptible to interference, noise is higher, when using capacitors as sensing elements, it is difficult to achieve high density and high-resolution sensing requirements.
	Piezoresistive	High sensitivity, fast response.	Difficult to preserve the materials, materials must be polarized before they can be used, the conditions of polarization treatment are very strict.
Flexible fiber optic sensor		High spatial resolution, no electrical interference, fast response, low cost.	Overall structure lacks flexibility, high dependence on elastomer.

**Table 5:** Characteristic parameters of data gloves.

Category of Sensor Technology	Main Materials	References
Flexible strain/pressure sensor	Silicone and carbon black	A textile glove [114]
	Mixture of Ecoflex 00-30 and carbon black nanoparticles	Data glove [120]
	Multiwalled carbon nanotube	Data glove [121]
	Silicone resin	A textile base glove [122]
	Piezoresistive yarn	Human–Machine Interface Glove [123]
	Elasticized fabric	CyberGlove [124]
	Polyamide/Lycra	SensoriGlove [125]
	NinjaFlex	Data glove [126]
	GO	Data glove [127]
	Silver nanoparticles–double covered yarn (AgNPs–DCY) composite yarn	An electronic data glove [128]
Flexible fiber optic sensor	Flexible grating strip	Data glove [129]
	Flexible grating strip	Data glove [130]
	Optical fiber	5DT Data glove [131]
	LED phototransistor	5DT Data glove [132]
	Flexible silicone rubber fiber	Data glove [133]
	Plastic multimode Fiber CK-20	Data glove [134]
	Optical fiber cable	VPL Data glove [135]

**Table 6:** Accuracy of different classifiers used to classify EMG.

Method of Classification	Recognition Accuracy	References
Nonlinear Logistic Regression (NLR)classifier	99%	[178]
Recurrent Neural Networks (RNN), Knowledge-based postprocessing model (Sequential decision algorithm)	4% better than the others	[179]
Fast Independent component Analysis (FastICA) algorithm	real-time decomposition 86% offline decomposition94%	[180]
scaled conjugate algorithm Levenberg algorithm	96.8% 98.8%	[181]
Trbaggboost algorithm	97.04%	[182]
ANN	83%	[183]
LDA	able-bodied 95% amputee 87%	[184]
SVM	87.88%	[185]
	95.32 ± 1.35%	[186]

**Table 7:** Related commercial applications.

Application Field		References
Gesture language recognition	Translate gesture language to voice or text.	[153,187,188,189,190,191,192]
Human–computer interaction	Virtual Reality Game Control.	[193,194,195]
	Surgical training.	[196,197]
Robot control	Control of prosthesis.	[198]
	Rehabilitation training.	[6,199,200,201]
	Soft gripper.	[202,203,204,205,206]

## Data Availability

The data presented in this study are available upon request from the corresponding author.
